# Fentanyl, Heroin, Methamphetamine, and Cocaine Analyte Concentrations in Urine Drug Testing Specimens

**DOI:** 10.1001/jamanetworkopen.2024.41063

**Published:** 2024-10-24

**Authors:** Andrew S. Huhn, Penn Whitley, B. Levi Bolin, Kelly E. Dunn

**Affiliations:** 1 Department of Psychiatry and Behavioral Sciences, Johns Hopkins University School of Medicine, Baltimore, Maryland; 2 Millennium Health, San Diego, California

## Abstract

**Importance:**

The US is experiencing a protracted drug overdose crisis primarily associated with exposure to illicitly manufactured fentanyl (IMF), methamphetamine, and cocaine. Overdose risk and treatment responses may be directly affected by absolute drug exposure concentrations and drug use prevalence.

**Objective:**

To quantify changes in absolute drug exposure concentrations from 2013 to 2023.

**Design, Setting, and Participants:**

This cross-sectional study analyzed urine drug testing (UDT) results from urine specimens collected between January 1, 2013, and August 22, 2023, in 49 states and the District of Columbia. Urine specimens were obtained from patients aged 18 years or older who presented to substance use disorder treatment clinics. The UDT was ordered by clinicians based on medical necessity.

**Exposures:**

Urine specimens were analyzed for the following drugs or metabolites (analytes tested in parentheses): fentanyl (fentanyl), heroin (6-monoacetylmorphine), cocaine (benzoylecgonine), and methamphetamine (methamphetamine) using liquid chromatography with tandem mass spectrometry.

**Main Outcomes and Measures:**

Relative concentrations of fentanyl, heroin, cocaine, and methamphetamine. Creatinine-normalized drug concentration values were log-transformed prior to visualization and statistical analyses. The Mann-Kendall trend test was performed to examine trends over time. To estimate the geospatial and temporal patterns of drug concentration, a second series of models (1 for each drug) with an interaction effect for clinic location and collection year were fit.

**Results:**

A total of 921 931 unique UDT samples were collected from patients (549 042 males [59.6%]; median [IQR] age, 34 [27-44] years). The adjusted fentanyl concentration in urine specimens was 38.23 (95% CI, 35.93-40.67) ng/mg creatinine in 2023 and 4.61 (95% CI, 3.59-5.91) ng/mg creatinine in 2013. The adjusted methamphetamine concentration was 3461.59 (95% CI, 3271.88-3662.30) ng/mg creatinine in 2023 and 665.27 (95% CI, 608.51-727.32) ng/mg creatinine in 2013. The adjusted cocaine concentration was 1122.23 (95% CI, 1032.41-1219.87) ng/mg creatinine in 2023 and 559.71 (95% CI, 524.69-597.06) ng/mg creatinine in 2013. The adjusted heroin concentration was 58.36 (95% CI, 48.26-70.58) ng/mg creatinine in 2023 and 146.59 (95% CI, 136.06-157.92) ng/mg creatinine in 2013. Drug concentrations varied across US Census divisions.

**Conclusions and Relevance:**

This cross-sectional study found that absolute concentrations of fentanyl, methamphetamine, and cocaine in urine specimens increased from 2013 to 2023, with a decrease in heroin concentration during that period. The findings suggest that exposure to these substances, as well as the illicit drug supply, has fundamentally changed in many parts of the US, highlighting the need to reinforce surveillance initiatives and accelerate efforts to treat individuals with IMF and/or stimulant exposure.

## Key Points

**Question:**

Have concentrations of opioids and stimulants in urine specimens changed in the past decade during the twin opioid-stimulant overdose epidemic?

**Findings:**

In this cross-sectional study of 921 931 unique urine samples collected between 2013 and 2023, adjusted concentrations of fentanyl, methamphetamine, and, to a lesser degree, cocaine increased while heroin concentration decreased.

**Meaning:**

Findings of this study suggest that exposure to these substances and the illicit drug supply has fundamentally changed in the US over time, necessitating reinforced surveillance initiatives and accelerated efforts to treat individuals exposed to illicitly manufactured fentanyl and/or stimulants.

## Teaser

This cross-sectional study examines patterns of illicit or nonmedical drug exposure detected in urine samples from patients in substance use disorder treatment clinics across the US.

## Introduction

The US is facing a deadly and protracted drug overdose epidemic characterized by opioid and stimulant exposure.^1^ Illicitly manufactured fentanyl (IMF) is responsible for at least 75% of drug-related overdose deaths, and reports suggest that use of the stimulants methamphetamine^2^ and cocaine^3^ either independently or together with IMF^4^ is increasing nationally.^5^ Concurrent use of IMF and stimulants has been referred to as the fourth wave of the overdose crisis.^6^ Ethnographic reports suggest that some persons using IMF may intentionally also take stimulants to prolong euphorigenic effects and/or reduce the severity of other sequelae (eg, opioid withdrawal).^4,7^

These findings are particularly concerning given evidence that drug use patterns shifted during the COVID-19 pandemic from exposure to primarily heroin to more dangerous products. Data reveal that the COVID-19–related lockdowns rapidly altered drug trafficking routes to exacerbate a growing preference for products that can be synthesized locally; that is, it became cheaper and more efficient to manufacture and ship precursor chemicals for IMF rather than growing and harvesting the *Papaver somniferum* (poppy) plants necessary to refine and ship heroin.^8^ Supply-side motivation for IMF vs heroin production is not likely to shift in the near future. Use of drugs also generally increased in the US during the pandemic^9,10^ despite a marked reduction in the availability and/or increased cost of some illicit products, including commercially produced opioids,^11,12^ which is hypothesized to have prompted many individuals to shift from lower- to higher-potency products.^13^ Rates of drug overdose deaths were also substantially higher in the post– vs pre–COVID-19 eras.^14^ Higher-potency products confer severe health consequences, increase overdose risk, and engender more severe physical dependencies that can challenge treatment effectiveness compared with lower-potency products.^15^

A complete understanding of the potential clinical implications of changes in drug exposure—especially differences in the absolute exposure levels of IMF and stimulants—has been hampered by a lack of sensitive surveillance methods that permit quantitative analyses of opioid and stimulant use. Existing reports are derived primarily from evaluations of overdose deaths, drug testing programs, broad epidemiological assessments, or ethnographic surveys of persons who use drugs, all of which provide limited scope and are affected by regionally specific differences in reporting methods.^16,17^ Systematic, objective data regarding drug exposure are primarily obtained from wastewater analyses,^18,19,20^ but despite quantitative urine drug testing (UDT) for these substances having been performed in clinical practice for decades, population-level analyses of these data are lacking.

Herein, to quantify changes in absolute drug exposure concentrations from 2013 to 2023, we analyzed quantitative UDT data for fentanyl, heroin, cocaine, and methamphetamine from over 900 000 urine specimens collected in substance use disorder (SUD) treatment settings throughout the US. Samples were tested for the quantitative level of exposure.

## Methods

### Urine Drug Testing Data

This retrospective, cross-sectional study analyzed UDT results from urine specimens collected between January 1, 2013, and August 22, 2023, and tested by Millennium Health, a specialty laboratory serving health care practices. Urine drug testing was ordered by clinicians based on medical necessity. Analyzed urine specimens were obtained from patients aged 18 years or older who presented to SUD treatment clinics in 49 states and the District of Columbia. South Dakota was not represented in the analysis due to limited urine specimen volume in the state. Urine specimens were analyzed using a liquid chromatography with tandem mass spectrometry laboratory-developed testing method, and performance characteristics were identified by Millennium Health, which is certified by the Clinical Laboratory Improvement Amendments and accredited by the College of American Pathologists for high-complexity testing. The Aspire Independent Review Board approved the study protocol and waived the informed consent requirement because the study used deidentified data. We followed the Strengthening the Reporting of Observational Studies in Epidemiology (STROBE) reporting guideline.

The following drugs or metabolites were studied when ordered by a clinician (analytes tested in parentheses): fentanyl (fentanyl), heroin (6-monoacetylmorphine), cocaine (benzoylecgonine), and methamphetamine (methamphetamine). The analyses focused on illicit or nonmedical use only; UDT results associated with a reported prescription by the ordering physician were excluded. Quantitative UDT results were identified for a subset of analytes or metabolites, and concentration values were expressed as creatinine-normalized concentration values (ng drug/mg creatinine). Creatinine concentrations were identified photometrically using the Jaffe method.^21^

### Covariates

Additional characteristics for each urine specimen included patient sex and age (discretized into 5 groups: 18-24 years, 25-34 years, 35-44 years, 45-54 years, and ≥55 years), location of the clinic (9 major US Census divisions), and urine specimen collection year. Race and ethnicity could not be included due to high levels of missing data.

### Statistical Analysis

A single urine specimen per patient was selected based on the earliest collection date. This selection eliminated repeated measurements that would confound the analysis of concentrations due to possible resampling of the same individual at different time points within the window of detection of a drug or across multiple instances of drug use over time. Creatinine-normalized concentration values were log-transformed prior to visualization and statistical analyses. Descriptive statistics were calculated for concentration values of the 4 drugs of interest for each year of the study. A Mann-Kendall trend test was performed on yearly median concentration values to examine trends over time. Linear regression models for each drug were used to evaluate the implications of demographic features for drug concentration levels. Collection year, clinic location (US Census division), patient sex, and patient age were modeled as additive covariates. To estimate the spatial and temporal patterns of drug concentration, we fit a second series of models (1 for each drug) with an interaction effect for clinic location and collection year. Coefficients and marginal estimates (using the lsmeans, version 2.30-0, package in R) were reported along with 95% CIs for all linear models. Both coefficients and marginal estimates were transformed back into the original linear space of the response value to make interpretation more intuitive.

Statistical significance was established with 2-sided α levels set at *P* ≤ .05. Analysis was performed using R, version 4.0.3 (R Project for Statistical Computing).

## Results

### Characteristics of the Patient Population

A total of 921 931 unique UDT specimens were collected from 4122 unique SUD treatment clinics and submitted for testing between January 1, 2013, and August 22, 2023 (Table). The analyzed population was composed of 372 889 females (40.4%) and 549 042 males (59.6%) with a median (IQR) age of 34 (27-44) years. The Pacific (23.1%), East North Central (19.5%), and South Atlantic (16.9%) were the most represented US Census divisions. The proportion of the total number of urine specimens per collection year ranged from 6.6% in 2018 to 15.2% in 2015.

**Table.** Characteristics of Patients With Urine Drug Testing Specimens Collected in Substance Use Disorder Treatment Settings Between January 1, 2013, and August 22, 2023

Characteristic	Urine specimens, No. (%)
All patients	921 931 (100)
Sex	
Female	372 889 (40.4)
Male	549 042 (59.6)
Age, y	
Median (IQR)	34 (27-44)
18-24	158 327 (17.2)
25-34	315 051 (34.2)
35-44	220 825 (24.0)
45-54	137 200 (14.9)
≥55	90 528 (9.8)
US Census division^a^	
East North Central	179 741 (19.5)
East South Central	44 599 (4.8)
Mid-Atlantic	60 516 (6.6)
Mountain	110 459 (12.0)
New England	22 689 (2.5)
Pacific	213 397 (23.1)
South Atlantic	155 461 (16.9)
West North Central	91 466 (9.9)
West South Central	43 603 (4.7)
Collection year	
2013	101 850 (11.0)
2014	114 347 (12.4)
2015	139 980 (15.2)
2016	95 266 (10.3)
2017	62 287 (6.8)
2018	61 154 (6.6)
2019	72 288 (7.8)
2020	61 314 (6.7)
2021	63 988 (6.9)
2022	81 951 (8.9)
2023	67 506 (7.3)

^a^ East North Central: Illinois, Indiana, Michigan, Ohio, Wisconsin; East South Central: Alabama, Kentucky, Mississippi, Tennessee; Mid-Atlantic: New Jersey, New York, Pennsylvania; Mountain: Arizona, Colorado, Idaho, Montana, Nevada, New Mexico, Utah, Wyoming; New England: Connecticut, Maine, Massachusetts, New Hampshire, Rhode Island, Vermont; Pacific: Alaska, California, Hawaii, Washington; South Atlantic: Delaware, District of Columbia, Florida, Georgia, Maryland, North Carolina, South Carolina, Virginia, West Virginia; West North Central: Iowa, Kansas, Minnesota, Missouri, Nebraska, North Dakota, South Dakota (not represented); West South Central: Arkansas, Louisiana, Oklahoma, Texas.

### Distribution of Drug Concentrations in Urine

**Figure 1.**
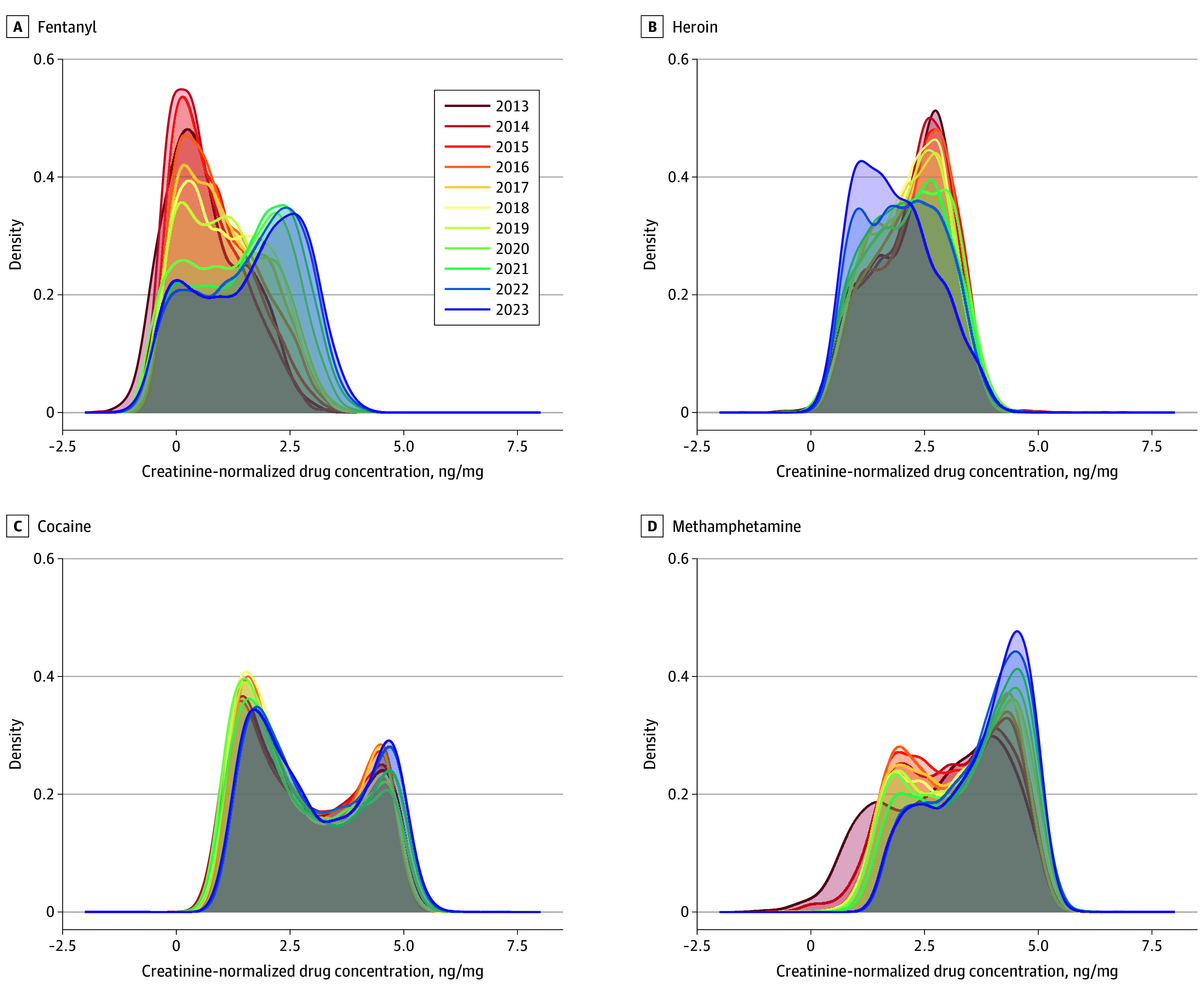
Distribution of Concentrations of Fentanyl, Heroin, Cocaine, and Methamphetamine in Urine Specimens From 2013 to 2023 Heroin (6-monoacetylmorphine) and cocaine (benzoylecgonine) are represented by the indicated metabolites. Fentanyl and methamphetamine were both measured as parent drugs. Concentration values are represented as log_10_-transformed values.

Figure 1 shows the distribution of creatinine-normalized concentrations of fentanyl, heroin, cocaine, and methamphetamine in urine specimens for each year of the study period. Fentanyl concentrations increased substantially, with median (IQR) values of 3.28 (0.06-1096.19) ng/mg creatinine in 2013 to 64.40 (0.04-18 666.26) ng/mg creatinine in 2023 (eTable 1 in Supplement 1). Methamphetamine concentrations also increased substantially during this period, from a median (IQR) of 1384.37 (0.07-659 442.72) ng/mg creatinine in 2013 to 10 028.06 (11.21-2 554 129.50) ng/mg creatinine in 2023. Similar but smaller-magnitude increases in exposure concentrations were also observed for cocaine, from a median (IQR) 372.79 (1.32-944 684.00) ng/mg creatinine in 2013 to 677.12 (7.00-2 197 625.88) ng/mg creatinine in 2023. In contrast, heroin concentrations decreased from a median (IQR) of 239.20 (0.21-2 329 440.00) ng/mg creatinine in 2013 to 54.91 (1.62-13 341.34) ng/mg creatinine in 2023.

### Changes in Drug Concentrations Over Time

**Figure 2.**
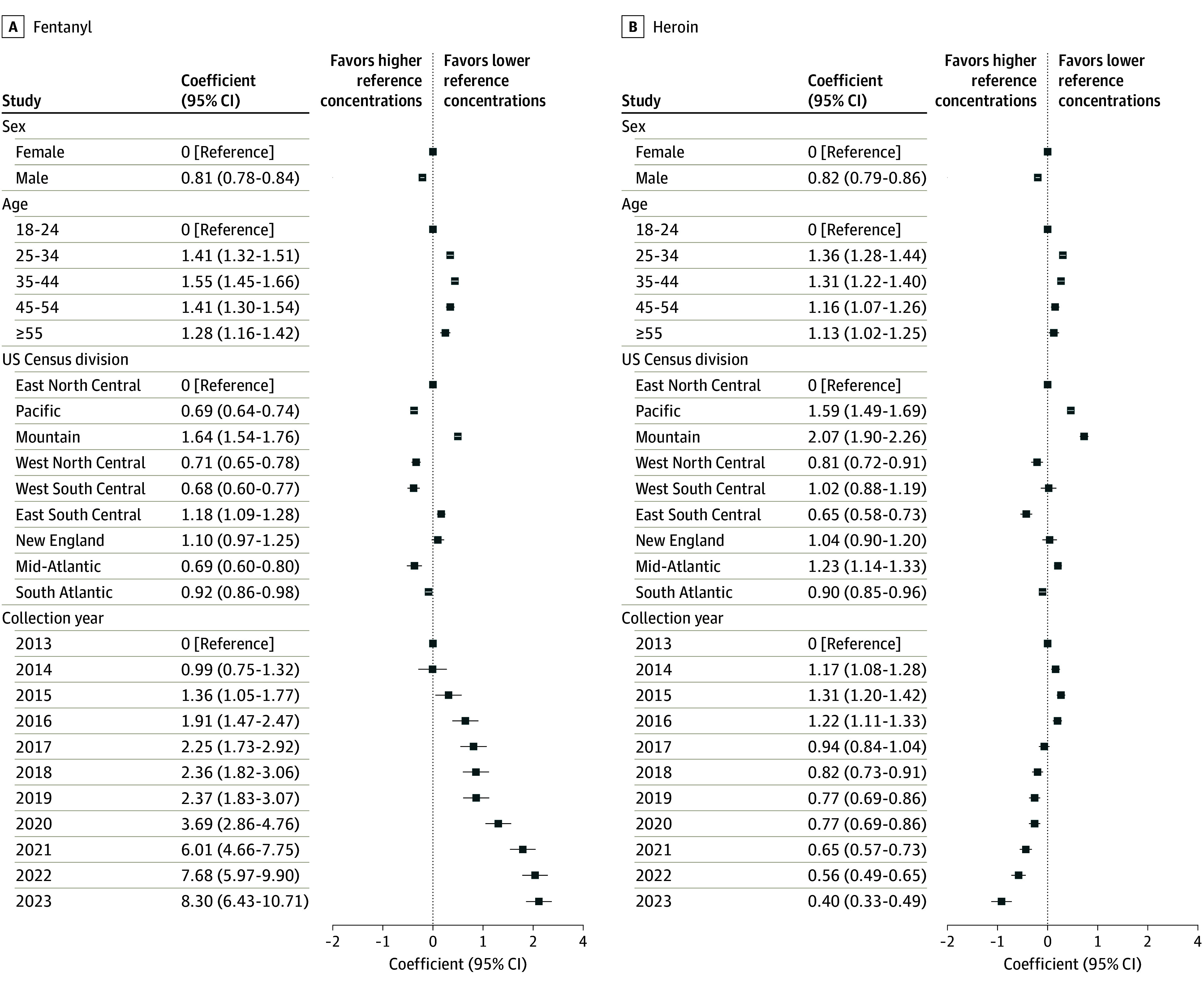
Coefficients for Additive Linear Regression Models of Fentanyl and Heroin Concentrations Linear regression models were performed on log-transformed concentration outcomes. Model coefficients and 95% CIs seen in tabular form were exponentiated to convert them to the original linear space of the concentration values and thus represent the fold-change between the given factor level and the reference level. Model coefficients shown in the plots are in the original log space.

**Figure 3.**
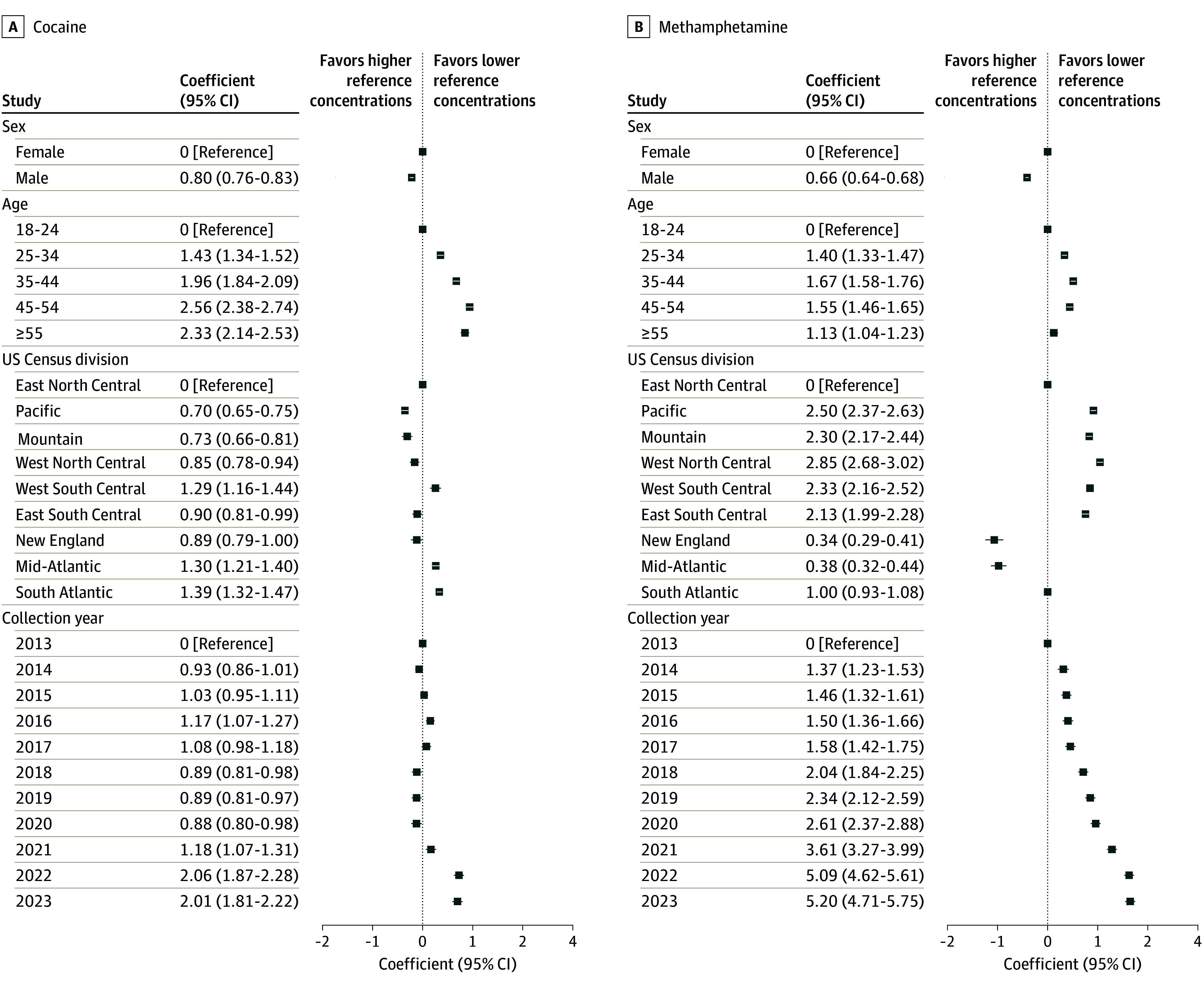
Coefficients for Additive Linear Regression Models of Cocaine and Methamphetamine Concentrations Models were performed on log-transformed concentration outcomes. Model coefficients and 95% CIs were exponentiated to convert them to the original linear space of the concentration values and thus represent the fold-change between the given factor level and reference level.

Drug concentrations in UDT changed significantly over time based on trend tests and regression results adjusted for demographic covariates (Figure 2 and Figure 3; eTables 1 and 2 in Supplement 1). Fentanyl and methamphetamine showed broad increases in concentration from 2013 to 2023. A Mann-Kendall trend test on median yearly concentrations showed that both fentanyl (τ = 0.964; *P* < .001) and methamphetamine (τ = 1.0; *P* < .001) increased consistently over the study period, and the incidence of positive UDT results for fentanyl and methamphetamine increased rapidly during most of the study period, peaking in 2021 (eFigure in Supplement 1).

The adjusted fentanyl concentration was 38.23 (95% CI, 35.93-40.67) ng/mg creatinine in 2023, which was 8.30 (95% CI, 6.43-10.71) times higher than the 4.61 (95% CI, 3.59-5.91) ng/mg creatinine concentration in 2013 (reference year). The adjusted methamphetamine concentration was 3461.59 (95% CI, 3271.88-3662.30) ng/mg creatinine in 2023, which was 5.20 (95% CI, 4.71-5.75) times higher than the 2013 concentration of 665.27 (95% CI, 608.51-727.32) ng/mg creatinine. The temporal pattern of change in cocaine concentration was more complex, with the adjusted 2023 concentration being 1122.23 (95% CI, 1032.41-1219.87) ng/mg creatinine, which was 2.01 (95% CI, 1.81-2.22) times higher than the 2013 level of 559.71 (95% CI, 524.69-597.06) ng/mg creatinine despite no upward or downward trend (τ = –0.0182; *P* > .99). Adjusted heroin concentration significantly decreased for much of the study period (τ = –0.745; *P* = .002), with the 2023 concentration being 58.36 (95% CI, 48.26-70.58) ng/mg creatinine, which was 0.40 (95% CI, 0.33-0.49) times lower than the 2013 concentration of 146.59 (95% CI, 136.06-157.92) ng/mg creatinine.

### Spatiotemporal Changes in Drug Concentrations

**Figure 4.**
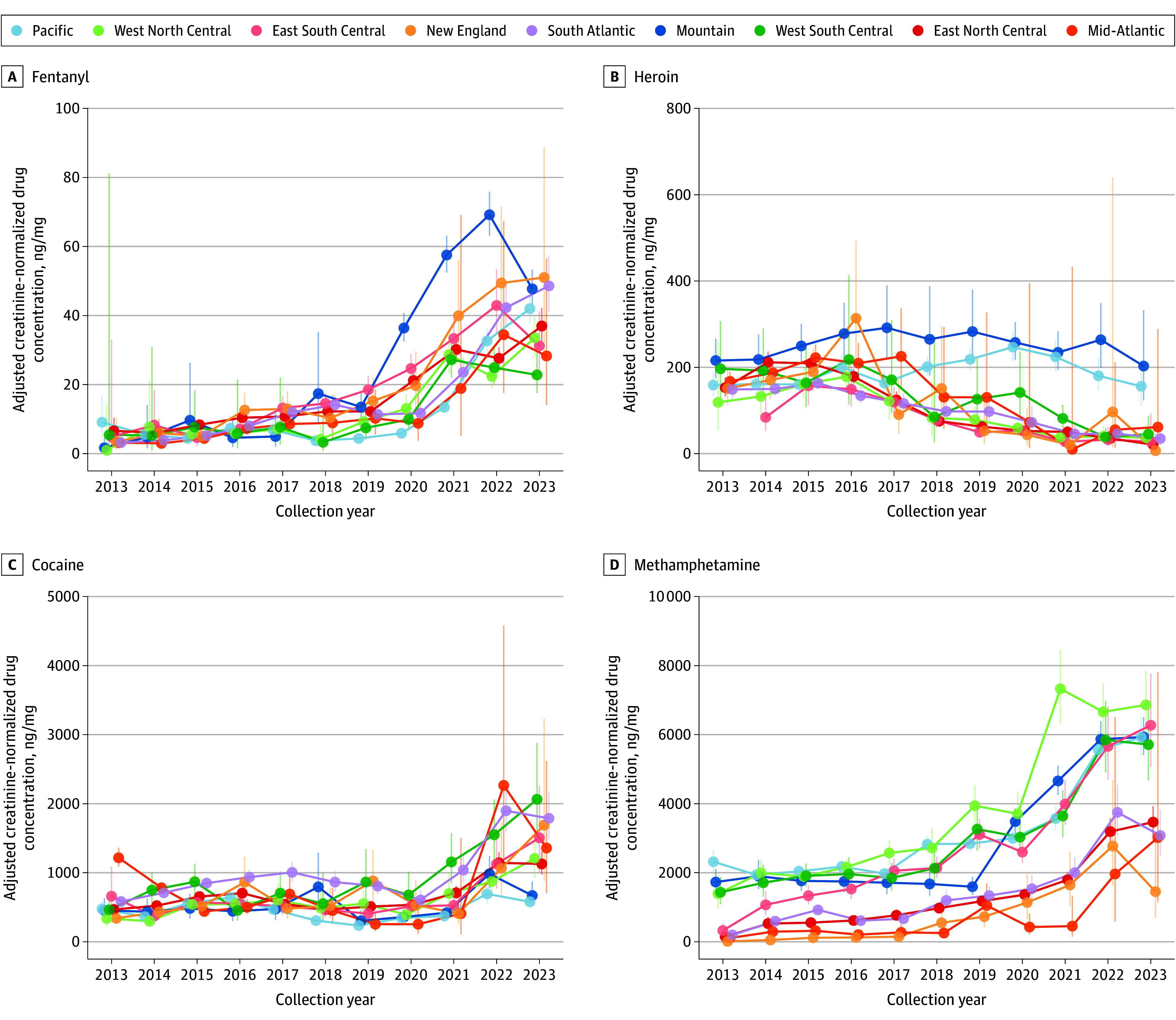
Spatiotemporal Patterns of Drug Concentration Change Adjusted mean concentration values were based on regression models with an interaction term for collection year and US Census division. Error bars represent 95% CIs.

Figure 4 and eTable 2 in Supplement 1 show spatiotemporal changes in creatinine-normalized drug concentrations over time by US Census division. Fentanyl had a relatively consistent temporal pattern across most regions, increasing between 2013 and 2023 for the nation. Fentanyl concentrations were higher in the Mountain division between 2020 (36.39; 95% CI, 32.56-40.66 ng/mg creatinine) and 2022 (69.21; 95% CI, 63.19-75.81 ng/mg creatinine) compared with other divisions before decreasing in 2023 (47.73; 95% CI, 42.77-53.27 ng/mg creatinine). National methamphetamine concentrations increased between 2013 (665.27; 95% CI, 608.51-727.32 ng/mg creatinine) and 2020 (1737.31; 95% CI, 1643.67-1836.28 ng/mg creatinine) before increasing even more rapidly between 2021 (2404.86; 95% CI, 2275.80-2541.24 ng/mg creatinine) and 2023 (3461.59; 95% CI, 3271.88-3662.30 ng/mg creatinine). Western Census divisions (Pacific, Mountain, West North Central, and West South Central) and the East South Central division demonstrated higher methamphetamine concentrations throughout the study period compared with the other divisions in the Eastern US. Cocaine concentration changes were relatively flat between 2013 and 2021 (660.98; 95% CI, 608.63-717.84 ng/mg creatinine) before increasing to an all-time high in 2022 (1155.78; 95% CI, 1069.80-1248.66 ng/mg creatinine). National heroin concentrations decreased between 2013 (146.59; 95% CI, 136.06-157.92 ng/mg creatinine) and 2023 (58.36; 95% CI, 48.26-70.58 ng/mg creatinine) for all regions except for the Pacific and Mountain divisions, wherein heroin concentrations were nearly as high in 2023 as in 2013.

## Discussion

The twin opioid-stimulant epidemic is the primary factor in drug overdose deaths in the US. This study found several key points that can guide public health efforts to intervene on overdose deaths and inform clinicians of more nuanced national and regional trends in drug use. While fentanyl-related overdose deaths increased year-over-year starting in 2013,^22^ on a national level, fentanyl dominated the illicit opioid supply starting in 2020. Most individuals with opioid use disorder (OUD) engaged in nonmedical use of commercially produced opioids prior to initiating heroin or IMF,^23^ and spatiotemporal trends in opioid prescribing and overdose deaths revealed that, while laws targeting pain management clinics were preventive of overdose deaths from natural or semisynthetic drugs, there were also unintended consequences—namely, increased rates of heroin- or IMF-related overdose deaths.^24^ Although fentanyl became common in the opioid supply over a decade ago, large increases of fentanyl and corresponding decreases of heroin in urine specimens were observed more recently.

The opioid drug supply in many regions of the US (eg, New England, Mid-Atlantic, and South Atlantic) consisted primarily of IMF at the time of this writing, which has implications for harm reduction and treatment strategies.^25,26^ Other areas, including the Mountain and Pacific regions, have seen a recent increase in fentanyl concentration in urine specimens while heroin concentration in urine samples was somewhat stable, suggesting that IMF is an additive rather than a replacement to heroin in those opioid supplies. Previous research on geographic patterns in drug overdose deaths found a complex interplay between socioeconomics and opioid prescriptions as primary factors in overall drug overdose deaths; for example, counties with high rates of poverty had higher overall rates of opioid prescriptions and overdose deaths, yet counties with higher educational and gross domestic product levels demonstrated sharper increases in the number of overdose deaths associated with a given number of opioid prescriptions.^27^ Data from UDTs could be combined with federal and/or prescriber data on opioid prescribing practices to identify regions that could most benefit from harm-reduction initiatives and a SUD treatment infrastructure. The clear and sustained increase in fentanyl concentration in urine specimens across the US suggests that IMF is the new normal for many individuals who use opioids, and national and regional patterns in fentanyl vs heroin concentrations in urine samples did not appear to have reached a steady state. Given the increased risk of overdose death^28^ and difficulties in OUD treatment for persons who use IMF,^29^ redoubling the expansion of drug surveillance efforts, such as the National Drug Early Warning System,^30^ may play a role in mitigation of the harms associated with IMF. In addition, platforms for crosstalk among clinicians nationwide may be associated with improved emergency and long-term medical care for persons with OUD.

This study also sheds light on increased stimulant use and related overdose deaths in the US; specifically, concentrations of methamphetamine in urine specimens increased steadily throughout the study period, with steep increases starting in 2020 in the West North Central, West South Central, Mountain, Pacific, and East South Central regions. This finding corresponds to increased methamphetamine use, risk behaviors associated with use, and methamphetamine-related overdose deaths.^2,31^ Concentrations of cocaine in urine specimens were somewhat stable between 2014 and 2020, with larger increases beginning in 2021, especially in the Mid-Atlantic, South Atlantic, West South Central, and New England regions. The majority of cocaine-related overdose deaths involved opioids, with an approximately 6-fold increase since 2015.^32^ Moreover, both methamphetamine and cocaine use are known to adversely affect OUD treatment outcomes; concurrent use of both opioids and stimulants is associated with increased drug craving, drug use, and treatment attrition.^33,34,35,36^ Individuals presenting to treatment with opioid-stimulant concurrent use might require additional services to attain a beneficial outcome. Additionally, given the increasing fentanyl adulteration in the stimulant supply in many areas of the US,^17^ drug-checking services might be particularly important for individuals who primarily use stimulants because inadvertent exposure to fentanyl in persons without opioid tolerance could precede an unexpected and fatal drug overdose.

Increases in fentanyl exposure present several unique challenges to persons with SUDs, potentially underlying reports of persons requiring more than 1 naloxone administration for overdose reversals,^37,38,39^ although this is not a universal phenomenon^40^ and is likely changed by concurrent use of other drugs. Increases in fentanyl concentration in urine specimens may also inform challenges in inducting patients from IMF onto buprenorphine, which has reportedly precipitated opioid withdrawal syndrome^29,41^ aversive enough that it reduced patients’ interest in future buprenorphine treatment.^42^ There are established micro-level and macro-level induction methods to transition individuals who use IMF onto buprenorphine maintenance,^43,44,45^ and buprenorphine remains an effective treatment for acute opioid withdrawal in emergency medicine.^46^ Individuals with SUD who regularly use IMF may require higher doses of buprenorphine or methadone, and efforts to expand access to both buprenorphine and methadone may reduce overdose risk and improve long-term outcomes for these persons.^26,47,48,49^

Although these consequences are often attributed to IMF’s increased potency compared with heroin,^50^ these data provide the first evidence that persons are presenting to treatment for SUDs with unprecedented concentrations of drug exposure. The pharmacokinetic profiles of these substances may partially explain outcomes as well. Therapeutically delivered fentanyl is known to be sequestered in peripheral fat and muscle stores and subject to a delayed, compartmental clearance process,^51^ although sequestration and clearance of supratherapeutic doses of IMF remain poorly understood. Methamphetamine (but not cocaine^52^) is also sequestered into peripheral organs^53^ and demonstrates extended urinary clearance patterns with accumulation after long-term dosing.^54^ Thus, the significant increases in fentanyl (by 8.30-fold) and methamphetamine (by 5.20-fold) concentrations observed in this study may reflect peripheral accumulation, changes in absolute concentration, or a combination of these phenomena. These data suggest that persons presenting to treatment with IMF, methamphetamine, or concurrent use likely have some level of peripheral accumulation, which independently predisposes them to health consequences, such as fentanyl- and methamphetamine-specific withdrawal syndromes, sensitization and delayed onset of respiratory depression after fentanyl exposure,^51^ and organ ischemia and hypertension associated with methamphetamine and cocaine exposures.

The findings of this study also support future efforts to leverage analyses of drug concentrations in urine samples that are being tested routinely as part of SUD treatment. Compared with existing surveillance methods (eg, wastewater testing, death certificate review, and ethnographic survey), aggregated quantitative UDT data offer a scalable surveillance method that can provide swift and nimble feedback on real-time changes in drug exposure levels as well as emerging public threats (eg, xylazine) that are not routinely captured by other methods. Efforts to quickly respond to a shifting drug supply can complement larger initiatives, such as the Stanford-Lancet Commission on the North American Opioid Crisis, which seeks to prevent the onset of nonmedical opioid use by promoting opioid stewardship among regulatory bodies and clinicians as well as to integrate evidence-based, equitable, and enduring SUD treatment into the US health care system.^55^

### Limitations

This study was limited by the observational nature of the data collection, which lacked experimental control over the time elapsed between last drug use and urine specimen collection. Additionally, UDT data cannot differentiate intentional from unintentional (eg, stimulants adulterated with opioids or vice versa) exposures, and concurrent use was not examined in these analyses. Furthermore, this study did not examine other drug use patterns that may be related to concurrent use or adulteration of the illicit drug supply, such as benzodiazepines or xylazine, but these factors are also associated with risk of drug overdose and have treatment implications. Moreover, individuals with SUD who were seeking treatment might differ in substance use patterns from individuals with SUD who were not seeking treatment.

## Conclusions

This cross-sectional study, to our knowledge, is the largest and most granular assessment of drug use patterns in the US to date. Its findings reveal changes in drug exposure and illicit drug supply over the past decade and highlight the need to reinforce surveillance initiatives and accelerate efforts to effectively treat IMF and/or stimulant exposure. The UDT results from 2013 to 2023 provide objective, biological evidence that persons presenting to treatment for SUD have increasingly higher absolute exposure to fentanyl and methamphetamine, as well as cocaine to a smaller degree, and decreasing exposure to heroin. These concentrations conformed to regional reports on drug use patterns, with IMF replacing heroin throughout most of the country and methamphetamine being most prominent in the Western regions. A concerted research strategy is necessary to lessen the consequences of increased exposure levels in individuals with SUD and to support focused public health and biomedical research efforts to understand and curtail overdoses from the twin opioid-stimulant epidemic.

## Notes

Supplement 1. **eTable 1.** Descriptive Statistics for Drug Concentration Distributions **eTable 2.** Adjusted Analyte Concentration for Cocaine, Methamphetamine, Heroin, and Fentanyl **eFigure.** Positive Urine Drug Screen Rate for Fentanyl, Heroin, Cocaine, and Methamphetamine from 2013 to 2023

Supplement 2.
Data Sharing Statement
