# Error in Figure 4

**DOI:** 10.1001/jamanetworkopen.2024.49821

**Published:** 2024-11-19

In the Original Investigation titled, “Fentanyl, Heroin, Methamphetamine, and Cocaine Analyte Concentrations in Urine Drug Testing Specimens”^1^ published October 24, 2024, there was an error in the Figure 4 key. “South Central” should be “East South Central.” The article has been corrected.

## References

[1] Huhn AS, Whitley P, Bolin BL, Dunn KE. Fentanyl, heroin, methamphetamine, and cocaine analyte concentrations in urine drug testing specimens. JAMA Netw Open. 2024;7(10):e2441063. doi:10.1001/jamanetworkopen.2024.41063 39446323 PMC11577146

