# Supplementary material for: Fentanyl, Heroin, Methamphetamine, and Cocaine Analyte Concentrations in Urine Drug Testing Specimens
Source: JAMA Netw Open. 2024 Oct 24;7(10):e2441063. doi: 10.1001/jamanetworkopen.2024.41063 (PMC11577146; doi:10.1001/jamanetworkopen.2024.41063)
Supplement: Supplement 1. — eTable 1. Descriptive Statistics for Drug Concentration Distributions eTable 2. Adjusted Analyte Concentration for Cocaine, Methamphetamine, Heroin, and Fentanyl eFigure. Positive Urine Drug Screen Rate for Fentanyl, Heroin, Cocaine, and Methamphetamine from 2013 to 2023 [file jamanetwopen-e2441063-s001.pdf]

Supplemental Online Content

Huhn AS, Whitley P, Bolin BL, Dunn KE. Fentanyl, heroin, methamphetamine, and cocaine analyte concentrations in urine drug testing specimens. *JAMA Netw Open*. 2024;7(10):e2441063. doi:10.1001/jamanetworkopen.2024.41063

eTable 1. Descriptive Statistics for Drug Concentration Distributions

eTable 2. Adjusted Analyte Concentration for Cocaine, Methamphetamine, Heroin, and Fentanyl

eFigure. Positive Urine Drug Screen Rate for Fentanyl, Heroin, Cocaine, and Methamphetamine from 2013 to 2023

This supplemental material has been provided by the authors to give readers additional information about their work.

© 2024 Huhn AS et al. *JAMA Network Open*

eTable 1. Descriptive Statistics for Drug Concentration Distributions

Analyte	Collection Year	mean	stdev	median	min	max	Mann-Kendall of Medians
fentanyl	2013	31.52	94.01	3.28	0.06	1096.19	tau=0.964, p=5.2e-05
fentanyl	2014	31.58	138.86	2.92	0.03	3172.00	
fentanyl	2015	52.88	207.61	4.46	0.11	4413.83	
fentanyl	2016	82.85	408.57	6.54	0.13	17908.20	
fentanyl	2017	97.43	319.45	8.27	0.19	5300.89	
fentanyl	2018	101.59	366.01	9.96	0.13	13981.75	
fentanyl	2019	115.43	371.98	10.73	0.10	9690.66	
fentanyl	2020	208.06	742.28	25.47	0.06	30738.00	
fentanyl	2021	307.68	862.13	47.85	0.06	17044.98	
fentanyl	2022	391.23	1757.59	57.72	0.03	143021.60	
fentanyl	2023	440.33	1123.69	64.40	0.04	18666.26	
heroin	2013	1499.98	41068.26	239.20	0.21	2329440.00	tau=-0.745, p=1.8e-03
heroin	2014	2385.32	98454.42	241.49	0.03	7234339.00	
heroin	2015	974.79	6640.70	267.84	0.35	425781.21	
heroin	2016	1162.16	23965.82	233.53	0.79	1620564.50	
heroin	2017	676.27	1831.83	169.06	0.58	58630.25	
heroin	2018	606.13	1382.83	175.29	0.05	25653.93	
heroin	2019	1342.26	35750.60	165.14	1.09	1704578.46	
heroin	2020	807.22	2428.37	177.65	0.28	80562.74	
heroin	2021	642.28	1668.30	131.82	0.69	35556.46	
heroin	2022	586.45	1596.70	101.63	1.64	33342.90	
heroin	2023	455.25	1189.35	54.91	1.62	13341.34	
cocaine	2013	13166.04	31761.69	372.79	1.32	944684.00	tau=-0.0182, p=1
cocaine	2014	12742.83	55731.26	360.75	1.11	4295659.00	
cocaine	2015	10638.39	26520.01	349.99	2.67	1454908.14	
cocaine	2016	11388.52	61734.03	384.44	3.21	5662354.93	
cocaine	2017	10418.96	22657.42	319.34	3.86	317904.71	
cocaine	2018	11799.86	65318.86	218.54	2.83	4115972.20	
cocaine	2019	26486.62	959265.05	234.40	2.86	76360799.00	
cocaine	2020	12426.64	31598.55	229.57	3.42	707288.23	
cocaine	2021	16241.06	37851.34	333.02	3.63	549511.36	
cocaine	2022	22033.64	145879.88	762.87	5.28	10489492.93	
cocaine	2023	20807.39	54889.14	677.12	7.00	2197625.88	
methamphetamine	2013	13646.78	34932.81	1384.37	0.07	659442.72	tau=1, p=2.6e-05
methamphetamine	2014	13943.29	31653.04	1597.75	0.37	411869.71	
methamphetamine	2015	14953.50	51966.08	1701.39	0.17	3772925.33	
methamphetamine	2016	15806.73	39461.27	2192.92	0.55	1970469.57	
methamphetamine	2017	14963.51	30778.33	2461.87	0.49	777446.82	
methamphetamine	2018	18284.87	37243.54	3178.67	0.34	705906.28	
methamphetamine	2019	23041.33	151901.95	3673.91	0.99	14392464.95	
methamphetamine	2020	22981.91	52034.62	4481.77	3.36	2457470.13	
methamphetamine	2021	27371.41	56653.24	6743.12	1.62	1584786.77	
methamphetamine	2022	30637.65	109840.42	9008.96	14.14	7830069.11	
methamphetamine	2023	29226.38	56178.42	10028.06	11.21	2554129.50	

© 2024 Huhn AS et al. *JAMA Network Open*

eTable 2. Adjusted Analyte Concentration for Cocaine, Methamphetamine, Heroin, and Fentanyl

Collection Year	Census Division	Fentanyl	Heroin	Cocaine	Methamphetamine
2013	U.S. Total	4.61 [3.59-5.91]	146.59 [136.06-157.92]	559.71 [524.69-597.06]	665.27 [608.51-727.32]
2014	U.S. Total	4.57 [3.97-5.26]	171.96 [161.64-182.95]	521.89 [493.57-551.83]	910.25 [847.27-977.90]
2015	U.S. Total	6.29 [5.76-6.87]	191.52 [181.26-202.35]	576.01 [546.90-606.68]	968.86 [915.91-1024.86]
2016	U.S. Total	8.80 [8.13-9.52]	178.54 [167.38-190.44]	652.55 [613.36-694.24]	998.82 [942.02-1059.04]
2017	U.S. Total	10.37 [9.48-11.33]	137.14 [126.20-149.03]	602.81 [559.58-649.37]	1048.87 [987.12-1114.48]
2018	U.S. Total	10.87 [9.98-11.85]	119.98 [110.10-130.75]	497.68 [459.83-538.64]	1354.26 [1275.64-1437.73]
2019	U.S. Total	10.92 [10.11-11.80]	113.07 [103.75-123.23]	496.00 [460.77-533.93]	1559.31 [1476.45-1646.81]
2020	U.S. Total	16.99 [15.92-18.12]	113.12 [103.50-123.64]	495.05 [454.99-538.62]	1737.31 [1643.67-1836.28]
2021	U.S. Total	27.70 [26.12-29.37]	94.86 [86.10-104.51]	660.98 [608.63-717.84]	2404.86 [2275.80-2541.24]
2022	U.S. Total	35.41 [33.52-37.40]	82.12 [72.43-93.10]	1155.78 [1069.80-1248.66]	3387.85 [3213.19-3572.01]
2023	U.S. Total	38.23 [35.93-40.67]	58.36 [48.26-70.58]	1122.23 [1032.41-1219.87]	3461.59 [3271.88-3662.30]
2013	East North Central	6.59 [4.20-10.34]	152.85 [132.73-176.02]	467.44 [397.21-550.09]	146.17 [101.66-210.18]
2014	East North Central	6.03 [4.42-8.23]	211.62 [189.83-235.91]	522.14 [454.48-599.88]	526.79 [389.08-713.24]
2015	East North Central	8.36 [7.39-9.46]	209.20 [193.96-225.64]	654.13 [597.03-716.70]	553.56 [460.83-664.96]
2016	East North Central	10.40 [9.39-11.53]	178.86 [163.11-196.14]	704.18 [639.27-775.68]	617.08 [504.41-754.93]
2017	East North Central	10.74 [9.40-12.28]	124.27 [107.69-143.42]	544.09 [480.10-616.61]	767.32 [629.15-935.82]
2018	East North Central	12.24 [10.87-13.78]	75.25 [63.55-89.11]	467.80 [419.82-521.27]	974.47 [848.31-1119.40]
2019	East North Central	12.21 [10.92-13.65]	62.49 [52.58-74.27]	509.79 [459.23-565.92]	1177.33 [1054.72-1314.19]
2020	East North Central	21.17 [19.13-23.42]	52.70 [43.60-63.69]	535.75 [474.87-604.44]	1365.13 [1219.60-1528.02]
2021	East North Central	30.18 [27.12-33.58]	50.09 [39.44-63.62]	709.16 [624.01-805.91]	1794.45 [1593.76-2020.41]
2022	East North Central	27.63 [24.81-30.76]	35.28 [24.75-50.29]	1143.12 [1007.38-1297.16]	3192.07 [2851.77-3572.97]
2023	East North Central	36.97 [32.43-42.14]	20.84 [13.38-32.45]	1127.47 [976.66-1301.57]	3465.89 [3063.37-3921.30]
2013	East South Central	4.88 [0.72-32.99]	NA [NA- NA]	659.33 [400.90-1084.34]	324.11 [198.77-528.47]
2014	East South Central	8.25 [3.70-18.42]	84.12 [52.98-133.57]	373.07 [265.45-524.32]	1069.95 [784.73-1458.83]
2015	East South Central	4.65 [2.36-9.13]	157.13 [113.65-217.24]	569.34 [449.87-720.54]	1334.25 [1092.09-1630.12]
2016	East South Central	7.12 [4.97-10.22]	149.25 [109.52-203.40]	554.22 [407.03-754.65]	1528.08 [1250.87-1866.72]
2017	East South Central	13.21 [10.53-16.56]	119.44 [92.24-154.65]	513.52 [396.74-664.67]	2057.32 [1762.02-2402.12]
2018	East South Central	14.53 [12.09-17.46]	77.37 [60.94-98.23]	462.84 [359.77-595.43]	2137.18 [1859.94-2455.75]
2019	East South Central	18.51 [15.31-22.37]	49.69 [37.21-66.34]	407.75 [317.21-524.13]	3106.41 [2720.62-3546.91]
2020	East South Central	24.67 [21.14-28.79]	53.11 [40.36-69.87]	513.05 [391.74-671.93]	2597.17 [2282.63-2955.05]
2021	East South Central	33.35 [27.49-40.45]	27.39 [17.48-42.92]	527.85 [388.71-716.81]	3990.09 [3399.04-4683.91]
2022	East South Central	42.93 [34.47-53.46]	32.39 [17.09-61.39]	1126.93 [773.99-1640.82]	5659.55 [4702.30-6811.66]
2023	East South Central	31.22 [23.65-41.22]	28.00 [8.47-92.58]	1505.29 [1003.86-2257.20]	6272.38 [5071.32-7757.91]
2013	Mid Atlantic	3.18 [1.58-6.38]	167.35 [148.07-189.15]	1216.24 [1090.95-1355.92]	108.62 [71.77-164.41]
2014	Mid Atlantic	2.97 [2.12-4.16]	187.40 [170.77-205.65]	782.03 [714.64-855.79]	292.61 [212.85-402.27]
2015	Mid Atlantic	4.40 [3.25-5.96]	222.26 [196.07-251.95]	440.09 [388.14-498.99]	317.51 [229.87-438.56]
2016	Mid Atlantic	7.27 [5.51-9.61]	209.44 [171.62-255.60]	499.71 [407.02-613.50]	206.96 [141.22-303.31]
2017	Mid Atlantic	8.59 [5.51-13.39]	225.48 [151.30-336.03]	690.51 [489.18-974.70]	272.14 [141.52-523.34]
2018	Mid Atlantic	8.91 [4.68-16.95]	130.78 [58.38-292.96]	451.92 [263.15-776.11]	252.29 [133.37-477.23]
2019	Mid Atlantic	10.22 [5.64-18.51]	130.33 [52.08-326.17]	255.33 [159.41-408.98]	1050.76 [654.79-1686.18]
2020	Mid Atlantic	8.77 [3.78-20.33]	72.63 [13.38-394.21]	253.78 [117.30-549.07]	426.32 [222.95-815.20]
2021	Mid Atlantic	18.86 [5.15-69.07]	9.86 [0.22-432.89]	407.64 [111.00-1497.06]	452.93 [154.26-1329.90]
2022	Mid Atlantic	34.45 [17.65-67.24]	55.47 [14.57-211.22]	2264.94 [1120.85-4576.82]	1964.11 [593.21-6503.12]
2023	Mid Atlantic	28.31 [14.19-56.45]	61.48 [13.13-287.94]	1357.88 [705.44-2613.74]	3017.83 [1167.20-7802.72]
2013	Mountain	1.68 [0.62-4.57]	215.70 [175.33-265.36]	454.32 [329.18-627.04]	1729.62 [1418.58-2108.85]
2014	Mountain	4.78 [1.63-13.99]	218.13 [172.84-275.30]	435.36 [306.55-618.30]	1899.47 [1527.39-2362.21]
2015	Mountain	9.66 [3.56-26.22]	249.53 [207.39-300.23]	482.82 [338.16-689.37]	1763.46 [1486.75-2091.67]
2016	Mountain	4.58 [1.98-10.62]	278.10 [221.63-348.94]	441.58 [303.00-643.54]	1747.70 [1441.19-2119.40]
2017	Mountain	4.97 [2.11-11.67]	291.54 [218.35-389.26]	474.77 [321.54-701.04]	1710.74 [1390.14-2105.29]
2018	Mountain	17.38 [8.58-35.19]	264.94 [181.44-386.88]	794.84 [492.10-1283.85]	1674.65 [1344.89-2085.27]
2019	Mountain	13.51 [10.48-17.41]	282.88 [210.78-379.65]	303.72 [204.55-450.98]	1594.36 [1359.20-1870.21]
2020	Mountain	36.39 [32.56-40.66]	257.63 [218.05-304.40]	359.45 [275.34-469.25]	3481.28 [3145.00-3853.52]
2021	Mountain	57.55 [52.50-63.07]	234.56 [194.34-283.11]	420.64 [332.43-532.25]	4659.06 [4260.27-5095.17]
2022	Mountain	69.21 [63.19-75.81]	263.85 [199.85-348.35]	983.74 [781.22-1238.75]	5866.75 [5392.93-6382.20]
2023	Mountain	47.73 [42.77-53.27]	202.88 [123.94-332.10]	669.99 [515.73-870.40]	5932.37 [5413.67-6500.77]
2013	New England	3.39 [1.12-10.22]	150.79 [114.27-198.98]	340.11 [260.09-444.75]	10.76 [5.69-20.36]
2014	New England	5.93 [3.48-10.12]	170.56 [127.20-228.70]	433.34 [327.14-574.02]	51.57 [23.16-114.87]
2015	New England	5.11 [3.66-7.12]	189.39 [147.46-243.26]	515.88 [405.08-656.98]	116.47 [68.81-197.15]
2016	New England	12.62 [9.07-17.54]	313.64 [199.51-493.06]	862.28 [606.05-1226.84]	124.66 [63.28-245.58]
2017	New England	12.96 [9.33-17.99]	90.35 [47.66-171.29]	480.47 [337.24-684.54]	147.97 [86.62-252.78]
2018	New England	10.32 [7.31-14.56]	150.98 [77.36-294.67]	518.73 [344.81-780.38]	550.26 [320.06-946.04]
2019	New England	15.26 [10.54-22.10]	52.78 [23.57-118.22]	880.95 [582.42-1332.50]	727.94 [426.10-1243.60]
2020	New England	19.71 [13.15-29.55]	43.64 [18.32-103.93]	532.81 [320.11-886.84]	1138.54 [662.19-1957.57]
2021	New England	39.95 [28.48-56.04]	20.13 [8.04-50.38]	407.81 [256.99-647.17]	1645.38 [1038.18-2607.70]
2022	New England	49.43 [34.20-71.44]	96.52 [14.57-639.59]	1066.78 [659.22-1726.31]	2773.16 [1642.94-4680.88]
2023	New England	51.03 [29.39-88.61]	6.85 [1.03-45.40]	1689.00 [884.90-3223.77]	1451.56 [695.40-3029.93]
2013	Pacific	9.09 [4.97-16.64]	158.84 [134.36-187.78]	475.27 [390.11-579.01]	2317.76 [2016.14-2664.49]
2014	Pacific	5.57 [3.40-9.12]	161.29 [138.73-187.53]	364.26 [307.97-430.84]	1938.39 [1747.07-2150.66]
2015	Pacific	4.43 [2.87-6.84]	160.19 [140.20-183.04]	554.19 [469.45-654.22]	2040.22 [1872.32-2223.18]
2016	Pacific	7.36 [5.02-10.79]	200.06 [175.28-228.34]	642.37 [536.84-768.64]	2176.89 [1999.30-2370.25]
2017	Pacific	6.87 [4.46-10.57]	163.26 [141.23-188.72]	470.36 [388.24-569.85]	1975.43 [1822.64-2141.03]
2018	Pacific	3.73 [2.68-5.19]	201.32 [180.52-224.51]	306.27 [245.45-382.15]	2827.49 [2619.06-3052.50]
2019	Pacific	4.40 [3.28-5.90]	218.51 [193.51-246.74]	235.28 [186.17-297.34]	2842.70 [2627.29-3075.77]
2020	Pacific	5.90 [4.85-7.16]	246.88 [214.87-283.66]	343.12 [257.33-457.51]	2995.71 [2723.05-3295.66]

© 2024 Huhn AS et al. *JAMA Network Open*

2021	Pacific	13.43 [11.72-15.38]	224.32 [191.57-262.68]	373.96 [288.31-485.06]	3570.04 [3236.94-3937.41]
2022	Pacific	32.66 [29.47-36.20]	180.36 [147.90-219.95]	692.55 [557.82-859.81]	5562.75 [5079.01-6092.57]
2023	Pacific	42.00 [37.76-46.71]	156.04 [112.39-216.64]	579.76 [475.77-706.49]	5923.08 [5404.07-6491.92]
2013	South Atlantic	3.19 [1.98-5.14]	148.70 [122.93-179.87]	583.48 [524.48-649.12]	198.14 [156.95-250.15]
2014	South Atlantic	3.86 [3.13-4.76]	149.97 [134.94-166.67]	709.80 [654.42-769.87]	595.46 [497.12-713.27]
2015	South Atlantic	5.22 [4.50-6.06]	163.38 [149.63-178.40]	848.77 [783.54-919.43]	923.96 [796.38-1071.97]
2016	South Atlantic	8.05 [6.91-9.38]	134.24 [117.34-153.56]	935.73 [839.97-1042.42]	611.47 [506.09-738.79]
2017	South Atlantic	11.99 [10.21-14.08]	116.30 [96.67-139.90]	1005.54 [874.49-1156.22]	667.26 [538.83-826.30]
2018	South Atlantic	14.17 [11.84-16.95]	97.80 [75.60-126.52]	863.71 [730.35-1021.43]	1199.04 [960.46-1496.89]
2019	South Atlantic	11.35 [9.58-13.43]	97.37 [76.67-123.65]	806.35 [693.68-937.32]	1337.85 [1119.96-1598.12]
2020	South Atlantic	11.74 [9.63-14.31]	72.43 [47.81-109.72]	605.68 [491.06-747.06]	1535.96 [1228.43-1920.48]
2021	South Atlantic	23.58 [20.02-27.76]	45.73 [32.46-64.42]	1038.02 [857.68-1256.27]	2001.02 [1635.13-2448.79]
2022	South Atlantic	42.30 [36.81-48.60]	46.30 [32.63-65.69]	1896.83 [1592.64-2259.12]	3747.79 [3093.53-4540.41]
2023	South Atlantic	48.60 [41.19-57.34]	34.74 [20.35-59.32]	1787.69 [1483.16-2154.75]	3079.07 [2477.24-3827.10]
2013	West North Central	0.95 [0.06-14.20]	118.82 [53.99-261.52]	337.74 [229.17-497.74]	1393.49 [966.27-2009.58]
2014	West North Central	7.74 [2.85-20.99]	132.65 [82.01-214.55]	297.78 [220.25-402.60]	1994.58 [1679.75-2368.42]
2015	West North Central	5.64 [3.62-8.80]	161.09 [128.61-201.79]	539.76 [439.52-662.86]	1888.52 [1701.42-2096.19]
2016	West North Central	5.85 [4.37-7.84]	178.20 [145.18-218.73]	547.05 [443.38-674.98]	2162.08 [1965.60-2378.20]
2017	West North Central	7.09 [4.71-10.68]	121.53 [79.38-186.07]	602.07 [427.41-848.11]	2572.28 [2245.11-2947.12]
2018	West North Central	4.16 [2.18-7.97]	82.75 [40.98-167.07]	489.50 [297.09-806.53]	2715.55 [2249.66-3277.93]
2019	West North Central	9.24 [6.97-12.23]	78.42 [56.13-109.57]	552.51 [415.19-735.24]	3942.89 [3438.35-4521.45]
2020	West North Central	13.05 [9.95-17.13]	59.28 [40.44-86.89]	392.79 [272.98-565.17]	3704.48 [3183.33-4310.95]
2021	West North Central	28.66 [23.31-35.23]	37.51 [26.27-53.55]	699.69 [490.81-997.47]	7327.96 [6348.11-8459.06]
2022	West North Central	22.39 [19.14-26.19]	39.95 [25.07-63.64]	872.95 [686.42-1110.18]	6659.47 [5931.62-7476.62]
2023	West North Central	33.54 [28.36-39.65]	36.77 [20.51-65.92]	1207.08 [935.64-1557.27]	6861.28 [6001.70-7843.97]
2013	West South Central	5.44 [0.36-81.15]	196.58 [126.17-306.27]	462.85 [342.16-626.13]	1433.41 [1097.67-1871.85]
2014	West South Central	5.25 [0.90-30.82]	192.41 [127.89-289.50]	748.95 [560.39-1000.94]	1709.93 [1314.72-2223.94]
2015	West South Central	7.64 [3.20-18.23]	163.99 [108.74-247.33]	868.47 [669.78-1126.10]	1910.57 [1611.35-2265.35]
2016	West South Central	5.83 [1.59-21.35]	217.82 [114.88-412.98]	454.84 [309.49-668.47]	1961.29 [1578.11-2437.52]
2017	West South Central	7.71 [2.71-21.96]	171.01 [94.70-308.84]	704.30 [487.95-1016.59]	1847.73 [1496.74-2281.02]
2018	West South Central	3.29 [0.85-12.69]	84.78 [27.10-265.18]	552.23 [305.73-997.47]	2129.60 [1665.33-2723.29]
2019	West South Central	7.46 [4.15-13.39]	126.23 [71.81-221.90]	864.84 [558.31-1339.65]	3263.06 [2648.06-4020.89]
2020	West South Central	9.89 [6.81-14.36]	141.66 [85.04-236.00]	677.57 [454.62-1009.87]	3030.88 [2500.04-3674.44]
2021	West South Central	27.20 [22.07-33.53]	81.51 [59.10-112.42]	1155.79 [852.64-1566.73]	3643.40 [3033.21-4376.33]
2022	West South Central	24.92 [20.42-30.40]	38.38 [25.20-58.46]	1549.91 [1167.39-2057.77]	5846.44 [4906.85-6965.95]
2023	West South Central	22.81 [17.61-29.54]	45.17 [23.61-86.43]	2062.32 [1477.14-2879.30]	5711.19 [4677.75-6972.96]

Adjusted mean (95% CI) concentration values are shown for each collection year and U.S. Census division. Predicted means are based on the model interaction term. U.S. Total concentration values are based on the collection year effect of the additive models.

© 2024 Huhn AS et al. *JAMA Network Open*

eFigure. Positive Urine Drug Screen Rate for Fentanyl, Heroin, Cocaine, and Methamphetamine from 2013 to 2023

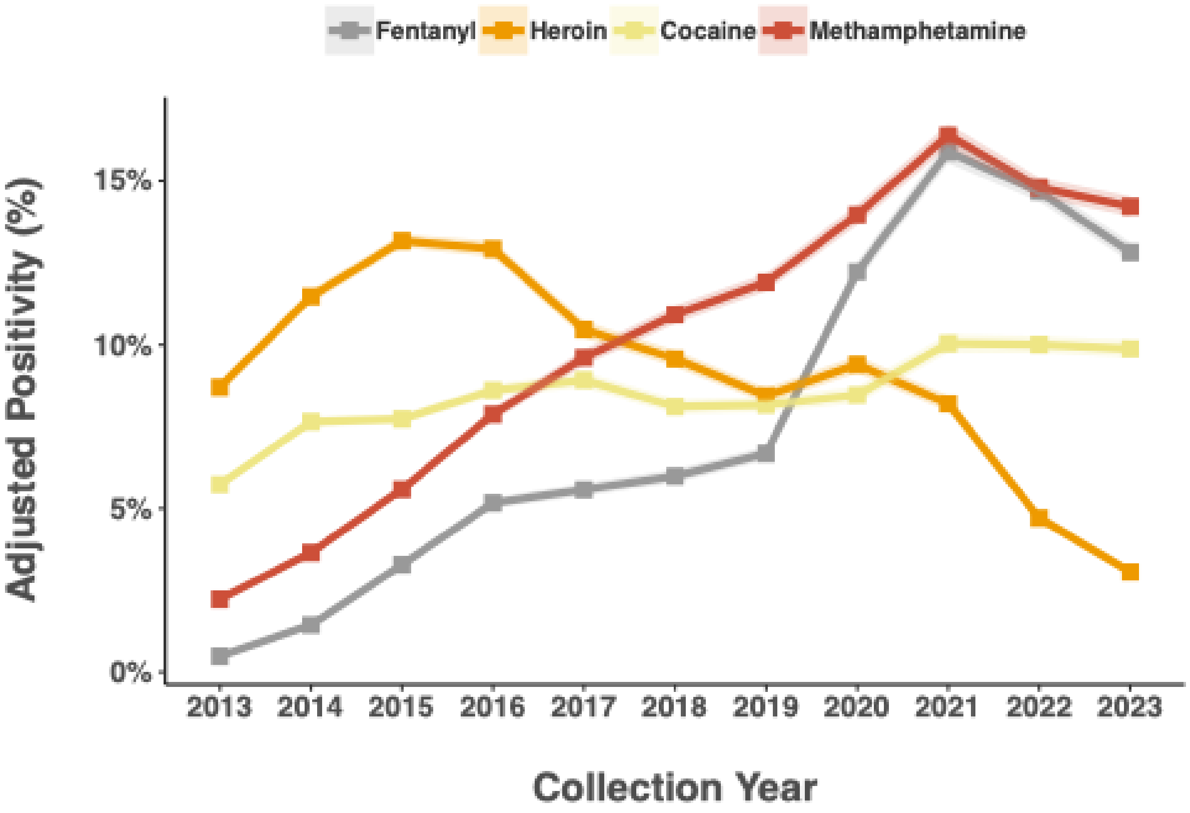

© 2024 Huhn AS et al. *JAMA Network Open*
