# Supplementary material for: Fentanyl, Heroin, Methamphetamine, and Cocaine Analyte Concentrations in Urine Drug Testing Specimens
Source: JAMA Netw Open. 2024 Oct 24;7(10):e2441063. doi: 10.1001/jamanetworkopen.2024.41063 (PMC11577146; doi:10.1001/jamanetworkopen.2024.41063)
Supplement: Supplement 2. — Data Sharing Statement [file jamanetwopen-e2441063-s002.pdf]

Data Sharing Statement

Data
Data available: No

Additional Information
Explanation for why data not available: The data analyzed in this study are owned by Millennium Health. Access to data may be available through a Data Use Agreement with Millennium Health.
